# A consistent long-lasting pattern of spatial variation in egg size and shape in blue tits (*Cyanistes caeruleus*)

**DOI:** 10.1186/s12983-018-0279-4

**Published:** 2018-10-03

**Authors:** Mirosława Bańbura, Michał Glądalski, Adam Kaliński, Marcin Markowski, Joanna Skwarska, Jarosław Wawrzyniak, Piotr Zieliński, Jerzy Bańbura

**Affiliations:** 1 Natural History Museum, Faculty of Biology and Environmental Protection, University of Łódź, Kilińskiego 101, 90-011 Łódź, Poland; 2 Department of Experimental Zoology and Evolutionary Biology, Faculty of Biology and Environmental Protection, University of Łódź, Banacha 12/16, 90-237 Łódź, Poland; 3 Department of Ecology and Vertebrate Zoology, Faculty of Biology and Environmental Protection, University of Łódź, Banacha 12/16, 90-237 Łódź, Poland

**Keywords:** Egg shape, Egg volume, Life history, Passerine, Spatial variation

## Abstract

**Background:**

Interspecies variation in avian egg shape and size is understandable in terms of adaptation, allometry and phylogeny. Within-species variation in egg properties influences offspring fitness and can be explained by differences in allocation of resources into reproductive components of life history in mulidimensionally variable environments. Egg size is inherently traded-off with clutch size, which may also be true of egg shape in some cases. We investigated long-term variation in egg shape and size between two geographically close populations of blue tits *Cyanistes caeruleus* in relation to clutch size and habitat differences.

**Results:**

The main finding is that there exists a persistent long-lasting pattern of spatial variation of egg size and shape between the two study populations of blue tits, 10 km apart, controlling for clutch size. Eggs in the urban park site were on average larger in volume and less spherical in shape than eggs in the forest site over 12 years of this study. Egg sizes were negatively associated with clutch sizes. Egg shape was not correlated with clutch size.

**Conclusions:**

Our findings suggest that the pattern of variation in egg size and shape results from different trophic richness of the breeding habitats of the study populations, demanding different allocation of resources and, especially, from the contrasting difference in the availability of calcium.

## Background

The avian egg is an evolutionarily elaborated version of the eggs of amniotes, in general, and the eggs of theropods, in particular [1]. In addition to containing the genetic equipment, it stores all the nutrients needed by the embryo to develop successfully [2]. At the level of class Aves, egg sizes are allometrically related to female body sizes, yet the relationship shows some differences between taxa and modes of development within birds [3]. Also the shape of bird eggs shows remarkable taxonomic diversity, with characteristic phylogenetically constrained patterns [2, 4, 5]. Inter-species variation in egg shape was found to be associated with avian flight adaptations [4], which, however, does not explain within-species variation. It seems reasonable to consider within-species variation in egg size as part of reproductive allocation strategy and seek its explanation within the framework of life-history theory [6].

Within species, different measures of nestling/fledgling performance, such as rates of growth and development, hatchability and chances to fledge, are usually positively affected by egg sizes [7, 8], at least to some threshold egg size, above which nestling performance increases no further [9]. In optimal environmental conditions birds would be expected to lay eggs of minimum size which still maximizes chances of nestling survival. Although producing eggs smaller than that size would be costly in terms of fitness, the negative effects can be overridden by parental care of nestlings, especially by adequate feeding [9, 10] which may be possible if the nestling stage coincides with the time of rich food abundance. If the amount of energy and nutrients allocated to a single egg affects not only its own size, but also the size of the subsequent eggs in a clutch, a trade-off between egg size and clutch size should arise because it is ultimately the number of surviving offspring which is the currency of fitness [6, 11, 12]. Optimal allocation of resources into individual eggs in the whole clutches is a key component of reproductive strategy that is certainly dependent on resource richness in the breeding habitat [6]. Because resources, including macronutrients and micronutrients, tend to be limited and variable in time and space (habitat), constraints on optimal allocation arise, and, therefore, some level of plasticity is favoured by natural selection [13]. Fitness may be locally maximised by different, resource-dependent allocation, resulting in producing clutches and eggs of different size in different habitats.

Egg shape is not usually considered in the context of intraspecies life-history variation, but it was hypothesised that optimal shape should depend on the number of eggs in the clutch in view of the way eggs are incubated [14]. Eggs of optimal shape should best fit the brood patch of incubating parents to be most efficiently maintained at an appropriate level of temperature for embryos to develop, resulting in clutches of different size having different optimal shapes of eggs [14]. In the case of larger clutches, it is not possible for all eggs to be in contact with the brood patch at the same time because they are distributed in layers within the nest cup and must be systematically rearranged to be uniformly warmed [15, 16]. If there is an optimal clutch-size-dependent shape of eggs, a pattern of relationship between egg shape and clutch size should be observable in avian populations because deviations from the optimal shape would be selected against [17]. In a study on fitness consequences of variation in egg shape in common blackbirds *Turdus merula* and great tits *Parus major* Encabo et al. [17] did not find any relationship between egg shapes and clutch sizes. This suggests that some other factors, perhaps limiting resources needed by females during the process of egg formation, should be taken into consideration. Calcium is a micronutrient whose availability is known to be often limiting for breeding birds during the stage of egg formation and nestling growth [7, 18–22]. Calcium availability seems to be able both to modify optimality criteria for egg shape and to generate its own selection pressures on egg shape and size.

The structure of eggshell is critically important for an avian embryo to develop normally into a hatchling that would have a chance to survive to fledging and then to the reproductive maturity [7, 22]. The shell must meet physiological functions associated with the embryo’s water management and gas exchange during incubation, which takes place in a nest containing the whole clutch. Hence it must be strong enough for eggs not to be damaged in the crush from incubating adults and other eggs. In fact, the shell must be produced quickly, in passerine in 1 day, and for just one egg at a time, because of egg fragility and bird mobility, with flight being especially prone to cause egg damage [2, 4]. In most small passerine birds eggshells are formed on the basis of the daily income of calcium, with no stored reserves available [7]. The availability of calcium may constrain a possibility of forming eggs of most profitable size in terms of fitness, which may generate selection pressures on the most compact and strong shapes. These factors may influence a balance between egg size and clutch size. In general, as economical a use of calcium as possible would be expected in calcium-poor habitats, where even defective eggshells are regularly recorded [18]. Calcium-poor habitats with otherwise good trophic conditions for breeding may generate selection for locally adaptive sizes and shapes of eggs as well as clutch sizes.

This study concerns blue tits *Cyanistes caeruleus* breeding in two areas that are contrastingly different in habitat properties, especially in terms of trophic conditions [23] and in terms of calcium availability [24]. One site is rich in caterpillars, the optimal food of nestlings, but poor in calcium, whereas the other site is poor in caterpillars, but rich in calcium [23, 24]. Bańbura et al. [24] revealed that eggs laid by blue tits in the calcium-poor area are on average smaller than in the calcium-rich area, with clutch sizes being larger in the former than in the latter. In this study we analyse a much larger dataset collected over 12 years of breeding. In particular, we focus on egg shape as well as on egg size. If the inter-habitat difference in egg traits resulted only from the association with clutch size, it should disappear after statistically correcting for variation in clutch size. If the difference remains after the adjustment, it must result from properties of the habitats compared. We expect that eggs should be not only smaller on average, but also more spherical in the calcium-poor area because round eggs make more economical use of calcium [14].

The aims of this study are to:

1. check if variation in egg volume between two spatially close populations of blue tits represents a consistent long-term pattern.
2. test if there exists any consistent pattern of variation in egg shape.
3. examine if variation in egg size and shape is associated with clutch size.

## Methods

### Study sites

This study was carried out between 2002 and 2013 as part of a long-term research project on the breeding biology of nestbox breeding populations of hole-nesting birds in two study sites within and near Łódź, central Poland. The study sites represent structurally different habitats of an urban parkland and a deciduous forest, 10 km apart. The urban park site (51°45’N; 19°24′E) is an 80 ha area that is composed of the zoological and botanical gardens, located in the SW part of the city of Łódź. The forest study site (51°50’N; 19°29′E) is a 130 ha area in the interior part of a mature mixed-deciduous forest (Łagiewniki Forest; 1250 ha in total), bordering on the NE suburbia of Łódź. The tree cover of the park area is highly fragmented and arranged to be useful for the purpose of animal and plant exposition, with trees constituting a mixture of many exotic and native species, deciduous and coniferous. This study site has a lot of open space, pathways, fences and buildings. Predominating tree species in the forest study area are pedunculate oaks *Quercus robur* and sessile oaks *Quercus petraea*. The tree canopy of this area is almost continuous and it also covers most of a small number of pathways crossing the forest.

Some characteristics of the study sites influence the availability of calcium for laying females. In the Łagiewniki Forest as a whole, including the study area, there has been a long-term tendency for water bodies and streams to dry up over the last 30–40 years. Water bodies in the urban park site are stabilised by artificial supply of water and, in addition, considerable parts of this area are watered as part of plant growing procedures. Soils of the Łagiewniki Forest are acidic, with pH < 5, whereas in the park site pH is higher (pH > 6) [25]. There are many artificial sources of calcium in the park area (lime, grit, buildings, pathways and so on), whereas such sources are lacking in the forest. The assemblage of shelled snails during the time of this study in the park site contained abundant synanthropic species, such as *Arianta arbustorum*, *Cepaea nemoralis*, and *Punctum pygmaeum*, which are completely absent from the forest [24]. Density of shelled snails is several times lower in the forest than in the park site [24].

Wooden nestboxes with a removable front panel [26] were erected on trees at a height c. 3 m above the ground level in both the study sites, c. 200 in the urban park site and 300 in the forest site. The nestboxes were distributed in a grid, keeping a distance of about 50 m between them. Mean density of nestboxes was similar in both study sites, 2.2–2.3 per 1 ha [27].

### Egg data

The field procedure in our study routinely starts in early spring (late March) from inspections of nestboxes to find signs of nest building and to determine nesting species. Then study sites are visited daily to record clutch initiation dates and clutch sizes in occupied nestboxes. Measurements of length and breadth of each egg in all clutches were taken with dial sliding calipers to the nearest 0.1 mm. Accidentally, eggs in a small fraction of clutches were not measured for technical reasons. This study is based on 8572 (3445 in the park site and 5127 in the forest site) eggs from 781 complete clutches (322 in the park site and 781 in the forest site) of blue tits measured over 12 years.

Based on lengths (L) and breadths (B) of individual eggs, volume (V) was calculated applying Hoyt’s [28] formula, V = 0.51 * L * B^2^ and shape (sphericity index, SH) was calculated according to the formula, SH = (B/L) * 100 [17, 29]. The B/L index is the reciprocal of the L/B index [29], thus indicating egg sphericity, which increases with egg breadth increasing in relation to egg length. These indices of individual egg shape and volume were data points analysed in this study.

### Statistical analyses

Because eggs in clutches tend to be similar in size to each other, their measurements cannot be treated as independent data records [30]. Accordingly, we calculated the intra-clutch repeatability of egg volume and egg shape for the whole data set, applying the intra-class correlation based on variance components obtained from one-way anovas [30], with standard errors estimated following Becker [31].

Egg volumes and shapes were analysed as dependent variables in separate linear mixed models in relation to year and site factors and clutch size as a covariate. Modeling started from models that included the first order interactions between all of the independent variables; non-significant interactions were deleted to leave the final model containing only significant interactions and all independent variables [32]. Clutch ID was included as a random effect in the models to control for clustering that resulted from eggs being laid in clutches (thus lacking independence from those in the same clutch), which was associated with degrees of freedom being estimated using the Satterthwaite method [33]. Statistical computing was performed using IBM SPSS Statistics 22 [33].

## Results

Both egg volume and shape show high variation between clutches and low variation within clutches, resulting in substantial repeatability (egg volume: *R* = 0.782 ± 0.027 (SE), F_780;7791_ = 40.1, *p* <  0.0001 and egg shape: *R* = 0.714 ± 0.026 (SE), F_780;7791_ = 40.1, *p* <  0.0001).

### Egg volume

**Fig. 1:**
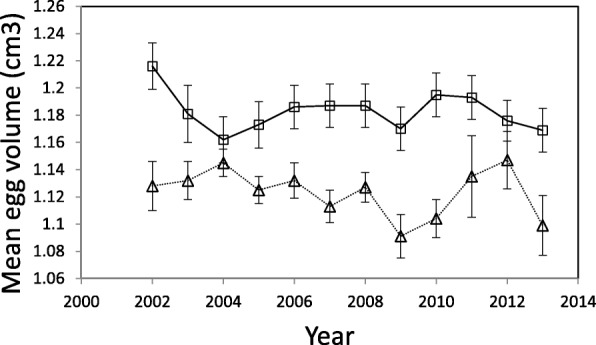
Mean volumes of blue tit eggs in the urban parkland site (squares) and the forest study site (triangles) during 2002–2013. Means ± standard errors are given

**Fig. 2:**
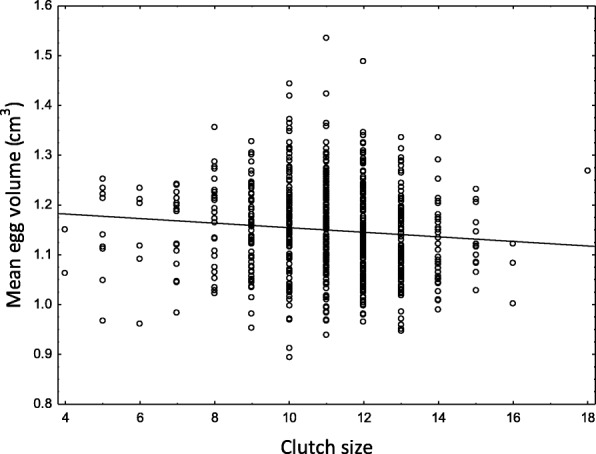
Relationship between the per-clutch mean egg volume and clutch size for 781 clutches of blue tits

The most striking result concerning egg volume in the study populations of blue tits was that the eggs showed a consistent pattern of variation between sites. Eggs in the urban park site were on average 5% larger than eggs in the forest site every year of the study (total mean volumes: 1.181 cm^3^ ± 0.005 (SE) v. 1.124 cm^3^ ± 0.004 (SE)). The data were modeled using two separate models. In the first model, which included year as categorical variable (12 years), neither effects of the two-way interactions nor of the year factor were significant (Table 1, Fig. 1). The site factor and the clutch size covariate had significant effects on egg volume, with the effect of clutch size being negative (Table 1, Fig. 2). The second model treated year as a continuous variable (Table 1). Because effects of the two-way interactions were non-significant in this model either, the main effects could be considered separately. While the effect of site remained highly significant, a significant negative effect of years was also revealed, with the clutch size covariate being non-significant (Table 1). This means that the eggs of the blue tits studied tended to reduce in size over time and that the tendency was consistently parallel in the two populations (Fig. 1).

**Table 1:** Summary of linear mixed models of egg volume of blue tits in relation to year, site and clutch size. Two separate models are shown: (i) with year as a categorical factor and (ii) with year as a continuous variable. Clutch ID included as a random effect

Effect	b-Estimate ± SE	df	F	P
Year as a categorical variable				
Final model				
Intercept	1.207 ± 0.022	1;883.5	3927.5	**< 0.0001**
Year	Multiple estimates	11;760.2	1.3	0.239
Site	−0.056 ± 0.006	1;762.7	71.7	**< 0.0001**
Clutch size	−0.0042 ± 0.0017	1;884.6	6.2	**0.013**
Deleted terms				
Site x Clutch size		1;753.9	1.3	0.330
Year x Site		11;739.9	1.3	0.212
Year x Clutch size		11;804.0	1.6	0.098
Year as a continuous variable				
Final model				
Intercept	5.306 ± 1.998	1;776.5	7.0	**0.008**
Year	−0.002 ± 0.0009	1;776.5	4.2	**0.040**
Site	−0.059 ± 0.006	1;777.8	87.1	**< 0.0001**
Clutch size	−0.002 ± 0.002	1;793.3	1.8	0.180
Deleted terms				
Site x Clutch size		1;782.9	1.2	0.259
Year x Site		1;776.9	1.9	0.164
Year x Clutch size		1;790.4	2.7	0.101

Bold entries highlight statistically significant effects, P < 0.05

### Egg shape

**Fig. 3:**
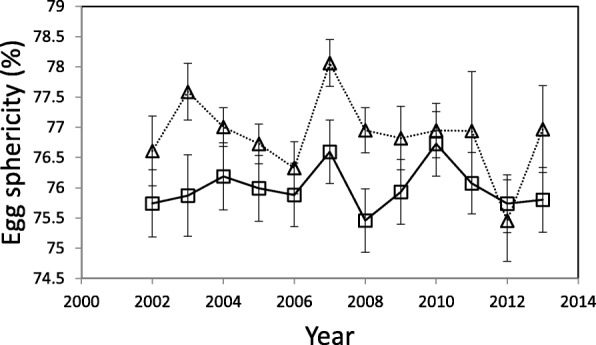
Mean shape indices of blue tit eggs in the urban parkland site (squares) and the forest study site (triangles) during 2002–2013. Means ± standard errors are given

Since the egg shape measure used in this study might not be independent of egg volume, the models examining egg shape included egg volume as a covariate in addition to year, site, clutch size and all two-way interactions. Two separate models were considered: the first one treated year as a categorical factor, while the second one included year as a continuous covariate (Table 2). Both final models showed a very similar pattern with a significant effect of the site – egg volume interaction (Table 2). This interaction in both the models resulted from egg sphericity being negatively correlated with egg volume in the forest site, but not in the urban park site (Table 2). The effect of site was not entangled in any interactions and, hence, was considered separately, showing a significant variation between the two study populations (Table 2, Fig. 3). Eggs were slightly more spherical in the forest population than in the urban park population (total mean sphericity: 76.992% ± 0.149 (SE) v. 76.026% ± 0.161 (SE), respectively). No differences in egg shape among years nor any trend over time were found (Table 2). Egg shape was not found to be dependent on clutch size (Table 2).

**Table 2:** Summary of linear mixed models of egg shape (sphericity) of blue tits in relation to year, site, clutch size and egg volume. Two separate models are shown: (i) with year as a categorical variable and (ii) with year as a continuous variable. Clutch ID included as a random effect

Effect	b-Estimate ± SE	df	F	P
Year as a categorical variable				
Final model				
Intercept	76.54 ± 1.03	1;1698.2	9859.9	**< 0.0001**
Year	Multiple estimates	11;764.9	1.5	0.118
Site	3.49 ± 0.96	1;8067.3	13.2	**< 0.0001**
Clutch size	0.014 ± 0.055	1;785.0	0.7	0.796
Egg volume	−0.68 ± 0.14	1;8484.1	21.6	**< 0.0001**
Site x Egg volume	−2.37 ± 0.81	1;8483.2	8.7	**0.003**
Deleted terms				
Site x Clutch size		1;757.4	0.001	0.999
Egg volume x Clutch size		1;8408.8	0.008	0.928
Year x Site		11;762.6	0.6	0.837
Year x Clutch size		11;771.9	0.9	0.562
Year x Egg volume		11;8299.4	1.2	0.284
Year as a continuous variable				
Final model				
Intercept	119.9 ± 65.0	1;775.6	3.5	0.061
Year	−0.021 ± 0.032	1;775.1	0.4	0.503
Site	3.57 ± 0.95	1;8113.6	13.9	**< 0.0001**
Clutch size	0.034 ± 0.053	1;795.1	0.404	0.525
Egg volume	−0.68 ± 0.13	1;8498.7	21.8	**< 0.0001**
Site x Egg volume	−2.41 ± 0.81	1;8495.3	8.9	**0.003**
Deleted terms				
Site x Clutch size		1;788.3	0.2	0.901
Year x Site		1;794.5	0.2	0.681
Egg volume x Clutch size		1;8374.0	0.695	0.405
Year x Egg volume		1;8455.2	1.9	0.163
Year x Clutch size		1;793.1	3.4	0.063

Bold entries highlight statistically significant effects, P < 0.05

## Discussion

This study found that egg shape as well as egg size may differ between spatially close populations of a small passerine. The main finding is that there exists a persistent long-lasting pattern of spatial variation of egg size and shape between the two study populations of blue tits, 10 km apart. Eggs in the urban park site were on average larger in volume and less spherical in shape than eggs in the forest site. Egg volume tended to decrease over the years in parallel between the urban park and the forest. We found no year-to-year variation in the case of egg shape.

Offspring size and number during particular breeding attempts as well as over the whole reproductive life of the individual are fundamental life-history traits [6]. The trade-off between egg size and clutch size in birds is a particular case of a more general pattern of trade-off between offspring size and number that is inevitable when the limited resources are allocated between individual offspring in a particular breeding attempt [6, 11, 34]. In wild bird populations living in heterogenous environments the negative relationship between egg size and clutch size expected from the trade-off may be masked by the effect of female (pair) body condition, where high condition females are capable of laying both big eggs and big clutches [35, 36]. The model of Charnov et al. [12] proposes that in the case of birds that produce relatively big clutches, such as tits (Cyanistes, Parus) or flycatchers (Ficedula), the masking effect may be weaker than in species laying clutches of smaller size. Accordingly, a significant negative correlation is usually found in species laying larger clutches [37–40]. In our study we also found such a significant negative correlation in blue tits.

Our study, based on individual eggs, controlling for clustering in clutches, considers egg shape (sphericity) as well as egg size (volume). Both egg volume and shape proved to be highly repeatable within clutches, which is typical of birds [24, 30]. Egg indices of both size (volume) and shape (sphericity) are derived from the basic linear measurements of eggs that are routinely taken in the field [28, 29]. Adamou et al. [41] have recently shown that these indices are good approximations of principal component measures of size and shape of eggs. However, in contrast to principal component indicators of shape, the measures of shape derived from egg linear dimensions, including the index of sphericity used in this study, may not be independent of egg volume. To control for this lack of independence, we used egg volume as a covariate in models explaining egg shape. The difference in average egg sizes between the blue tit populations nesting at the park site and the forest site for 2002–2009 was shown by Bańbura et al. [24]. Their analysis was based on per-clutch mean volumes and linear dimensions of a sub-set of the whole egg dataset. Our present findings more powerfully confirm the existence of a stable, long-lasting pattern of difference in egg volume and, in addition, in egg shape between the study populations inhabiting the urban park site and the forest site. We found that egg volumes significantly decreased over the years in a parallel manner in both the study sites. No consistent change over time was found in the case of egg sphericity.

Inter-annual, usually rather slight variation in egg size has been reported for many species of birds [41–43]. It was suggested that under current global warming, some trends in inter-annual variation in egg traits might be expected in different bird species [44], which in fact came true in several cases, yet sometimes trends were in an unexpected direction [45–47]. The latter is also the case in our present study, where we found a decreasing trend in egg volumes instead of an increasing trend that would be expected from earlier arguments [44]. In agreement with the global trend, air temperature in Poland, from the scale of the whole country to the local scale of our study area, is known to have been increasing over the last hundred years [48]. This increase manifests itself in both annual mean temperatures and in spring temperatures, resulting, however, not only in warming, but also in more frequent and less predictable extreme weather events [48], which may not be favourable to breeding birds. As far as we know, no data on inter-year variation in egg traits of blue tits are available in the literature, whereas there are a few reports concerning different populations of another parid species, the great tit. Jarvinen and Pryl [49] found no differences between years in egg volumes or linear dimensions in a south Finland population of this species. Slight inter-year variation in egg size and shape, entangled in interactions of the year factor with other factors, was reported by Ojanen et al. [50] for north Finland. Hõrak et al. [51] found significant year-to-year variation in egg volume and shape, with no clear pattern, in rural and urban populations of great tits in Estonia; a significant difference in egg volume between 2 years was also shown by Mӓnd et al. [52] in the same country. The differences most probably result from effects of year-to-year differences in ecological conditions prevailing during the time of egg formation on resource allocation between egg size and clutch size [9, 46, 53].

Spatial variation in egg volume and linear dimensions was analysed on the scale of entire Europe in the case of great tits, but not blue tits [54]. Small-scale spatial or habitat effects were also more often studied in great tits. Hõrak et al. [51] showed that eggs in an urban park site in Tartu, Estonia, were on average smaller than in a rural forest site (the distance between the study sites c. 5 km). By contrast, Riddington and Gosler [55] found that great tit eggs in Oxfordshire village and urban gardens were heavier (larger) than in the forest habitat of Wytham Wood (the distance between the gardens and Wytham Wood c. 2 km). Mӓnd et al. [52] reported that eggs in the deciduous forest site tended to be larger than eggs in the coniferous forest site in Estonia (the study sites to c. 10 km apart). Hargitai et al. [56] discovered that eggshells in an urban park were thicker than in a woodland site in great tits (the distance between the sites c. 20 km). Slight variation in egg size [57] and shape [58] was shown to be related to the altitude of nest sites in ultramarine tits (African blue tits) *Cyanistes teneriffe ultramarinus* in Algeria. In our study populations we previously found a significant between-habitat difference in mean egg sizes of blue tits, but no of great tits [24]. This difference in the patterns of egg size and shape variation between the tit species is confirmed by the present study on blue tits and our new data on great tits (in preparation). A very similar pattern of inter-habitat variation in egg volume was revealed for the same tit species in Burgundy, with no difference in great tits and a significant difference in blue tits, and with eggs being larger in urban habitats than in the forest (the study sites 40–100 km apart) [59]. Apart from our study, we are not aware of any other results describing clear patterns of variation in egg shape as well as egg size between habitats in European blue tits. The persistent difference in egg size and shape between sites with parallel fluctuations across the years of the study suggest that there exists a long-lasting difference between the sites, on the one hand, and a cause of year-to-year variation which is common for the two sites, on the other hand.

The basic idea behind our study system was to encompass study sites of contrasting habitats to be studied in a long-term perspective. As a consequence, we established our nestbox study areas in a large urban park habitat and in an interior part of a deciduous forest, assuming that the former would represent a sub-optimal habitat, while the latter would be the optimal habitat for nesting tits. The breeding density of blue tits is variable over the years, and with the grand mean values of 3.4 pairs/10 ha in the forest and 3.8 pairs/10 ha in the park, it is relatively low in comparison with the values typical of West European populations of this species [40], which is also true of great tits (own data). We estimate that 90–95% of breeding pairs of both tit species nests in nestboxes, even if both study sites are also rich in natural holes. Thus, the potential nest sites are superabundant. Of the available nestboxes, no more than 35% are occupied by all hole-nesting species in the forest site, with the corresponding figure for the park site being 60%, still leaving many holes free. The age structure seems not to much differ between the forest and the park populations – the proportion of the first-year adults is 41–44% against 56–59% of older adults in both the sites (own data, in preparation). The blue tits are typically single-brooded, with very rare, exceptional second breeding attempts.

In terms of the abundance of leaf-eating caterpillars, as key food for nestlings, the contrast between the forest and the urban park study sites is considerable. The leaf-eating caterpillars are on average three times more abundant in the forest site than in the park site, which leads to the corresponding difference between the sites in their suitability for rearing nestling tits [23, 27, 60, 61]. It is well known that tits and some other insectivorous species lay larger clutches in optimal habitats than in suboptimal ones because clutch size tends to be adjusted to the trophic conditions during the chick-rearing phase [9, 62–65].

In accordance with the difference between our study sites in the abundance of caterpillars, clutches of blue tits are on average larger in the forest site than in the urban park area, with some variation between years occurring [23, 60]. In fact, it is not only the abundance of leaf-eating caterpillars that differs between the two study sites. Insects and other arthropods in general are distinctly more abundant in the forest site than in the urban park site [66, 67], which creates more favourable trophic conditions for breeding insectivorous birds. On the other hand, the forest site is characterised by a five to six times lower density of shelled snails than the urban park site, with the latter area being home for abundant human-associated snails, such as *Cepaea* spp. [24]. It was shown by many authors that poor availability of shells and other sources of calcium is limiting for females during the time of egg formation, when the demands for calcium are highest [18, 19, 53, 68]. We suggest that this is also the case with blue tits in our forest study site. High availability of insect food and poor availability of calcium may maintain a pressure on females to move a balance in resource allocation towards producing smaller eggs because any potential initial disadvantages can be compensated for at the nestling stage. When considered separately in field experiments on blue tits in Scotland, the quality of supplemental food had positive effect, whereas supplemental calcium had no effect on egg sizes [69, 70]. Obviously, the experimental supplements used by Ramsay and Houston [69, 70] were transient factors, while the trophic characteristics of our study sites are enduring factors, both of which can have effects on egg size but at a different level. Transient factors may result in a release from trophic limitation, when supplementary food and/or micronutrients are provided, or in the limitation becoming even more severe, when the resources are reduced. Enduring factors generate selection pressures for economical use of resources, which can run adaptive changes in female physiology and oviduct morphology [4], resulting in producing smaller ova, needing less calcium. Transient factors would be expected to determine rather eggshell thickness than egg size or shape.

The difference in clutch size between our study sites may suggest that smaller mean egg sizes in the forest site than in the urban park site could potentially result just from different allocation of resources. However, we excluded such an effect in statistical analysis by including clutch size as a covariate in the models. Thus, the persistent difference in egg sizes between the study populations was shown to be independent of clutch size, which suggests that blue tits in the forest site lay smaller eggs than expected from their clutch size and from the respective trade-off between egg size and clutch size in comparison with the urban park site. Eggs laid in the forest site are not only smaller than eggs in the park site, but also tend to be more round, with roundness being the most calcium-saving shape of eggs [14]. Moreover, the eggs tend to become less spherical with the increasing volume in the forest, while no such tendency occurs in the park.

In contrast to egg sizes being related to clutch size, we found no correlation between egg shape and clutch size. We expected that such a correlation should occur in blue tits. Because blue tits lay some of the largest clutch sizes of any passerine [40] and a clutch in our study populations is on average composed of over 11 eggs [27], the eggs must be arranged in layers within the nest cup. As a consequence, to expose all eggs of a clutch to an appropriate temperature during incubation, females rotate and rearrange them regularly to enable them to be uniformly warmed by the brood patch, to allow air to circulate around eggs, and to dissipate heat when the eggs are too warm [14, 15, 17, 71, 72]. The physical shape, considered along the roundness-elongation axis, may directly influence feasibility of getting a space-saving arrangement of eggs within the whole clutch, which should be also affected by clutch size [14, 17]. It seems reasonable to expect that from the point of view of the space-saving three-dimensional arrangement of eggs in the space of nest cup, larger clutches should contain more elongated eggs. However, the acts of rotation and rearrangement of eggs by incubating females may expose eggs to elevated risks of breakage. They may be greater with increasing clutch size and with declining calcium availability. On the other hand, more spherical eggs form a stronger structure for a given, limited amount of calcium [14]. All this may account for our results that eggs in the forest site are more spherical than eggs in the urban park site and that it happens at least in some years that egg sphericity is positively related with clutch size. Analogously, Kouidri et al. [58] found that egg sphericity in North African ultramarine tit tended to increase with clutch size at high altitude, where ecological conditions were harsh. Gosler et al. [73] found that great tit eggs were more spherical in calcium-poor surroundings.

Thus, the patterns in egg shape variation seem to complement the pattern of variation in egg sizes in the study populations of blue tits. They both support the hypothesis that the availability of calcium may be the most important factor that affects variation in egg size and shape, resulting in the existence of stable spatial patterns. Under poor calcium availability, females would be expected to lay smaller and more spherical eggs than under rich calcium availability.

## Conclusions

We found in this study that both egg size and shape show consistent patterns of variation between two spatially close populations of blue tits. The difference in egg volumes between the two sites goes beyond the difference expected from the trade-off with clutch size, which is also true of egg shape. Overall, the patterns of variation in egg traits we found and their stability over time suggest that there may be different optimal sizes and shapes of eggs between the caterpillar-rich-calcium-poor habitat and the caterpillar-poor-calcium-rich habitat.
